# Nonclinical Safety Profile of Revusiran, a 1st-Generation GalNAc-siRNA Conjugate for Treatment of Hereditary Transthyretin-Mediated Amyloidosis

**DOI:** 10.1089/nat.2019.0796

**Published:** 2020-01-28

**Authors:** Jessica E. Sutherland, Julia L. Hettinger, Amy Chan, Jason Gilbert, Garvin L. Warner, Wendell P. Davis

**Affiliations:** Alnylam Pharmaceuticals, Inc., Cambridge, Massachusetts.

**Keywords:** siRNA, short interfering RNA, revusiran, hATTR amyloidosis, toxicity

## Abstract

Revusiran is a 1st-generation short interfering RNA targeting transthyretin conjugated to an *N*-acetylgalactosamine ligand to facilitate delivery to hepatocytes via uptake by the asialoglycoprotein receptors. Revusiran, in development for the treatment of hereditary transthyretin-mediated amyloidosis, was discontinued after an imbalance in deaths in the “ENDEAVOUR” phase 3 clinical trial. Nonclinical safety assessments included safety pharmacology, acute and repeat-dose toxicity, genotoxicity, and carcinogenicity. There were no effects on cardiovascular or respiratory function in monkeys after single doses of up to 100 mg/kg. No neurological effects were noted in monkeys in repeat-dose studies up to 300 mg/kg. Revusiran was well tolerated in repeat-dose mouse (weekly doses) and rat and monkey (five daily doses followed by weekly doses) toxicity studies. The no observed adverse effect level (NOAEL) in rats was 30 mg/kg based on reversible microscopic changes in liver that were accompanied by correlating elevations in clinical chemistry at higher doses. Dose-limiting toxicity was absent in monkeys, and the NOAEL was 200 mg/kg. There was no evidence of genotoxicity *in vitro* or *in vivo* at limit doses or carcinogenicity in a 2-year study in rats at doses up to 100 mg/kg. Overall, these results demonstrate that revusiran had a favorable nonclinical safety profile.

## Introduction

RNA interference is a naturally occurring cellular mechanism for regulating gene expression by which a double-stranded short interfering RNA (siRNA) (typically 21–23 nucleosides in length) mediates sequence-specific degradation of messenger RNA (mRNA), leading to the reduced synthesis of the corresponding protein [1]. When synthetic siRNAs are introduced into cells, the guide (or antisense) strand of the siRNA loads into an enzyme complex called the RNA-Inducing Silencing Complex (RISC). This enzyme complex subsequently binds to its complimentary mRNA target sequence, mediating its cleavage by argonaute-2 endonuclease (Ago2) and thereby preventing synthesis of the target protein [2,3].

Large-molecular-weight (16 kDa), negatively charged siRNA molecules cannot enter cells without a delivery agent [4]. One approach is to formulate siRNAs in intravenously administered lipid nanoparticle formulations that preferentially distribute to hepatocytes via an apolipoprotein E (ApoE)-dependent process mediated by low-density lipoprotein and other ApoE-binding receptors [5].

Another approach utilizes siRNA that is conjugated to a trimer of *N*-acetylgalactosamine (GalNAc) residues that are recognized by and transported into hepatocytes by asialoglycoprotein receptors (ASGPRs) located on the surface of hepatocytes [4,6]. Once the ligand-receptor complex is internalized, the cargo is released into the endocytic pathway with subsequent engagement with the RISC complex; the ASGPR is then rapidly recycled to the cell surface, thus enabling multiple rounds of cargo uptake and release.

Revusiran is a chemically synthesized double-stranded siRNA (directed against transthyretin [TTR] mRNA) that is covalently linked at the 3′-end of its sense strand to a ligand containing three GalNAc residues. The sense and antisense strands contain 21 and 23 nucleosides, respectively, and form a 21-mer duplex with a 2-base overhang at the 3′-end of the antisense strand. The nucleosides in each strand are connected through 3′–5′ phosphodiester linkages, thus forming the sugar-phosphate backbone of the oligonucleotide.

The antisense strand contains two consecutive phosphorothioate linkages at its 3′-end that improve its resistance to exonuclease degradation [7]. Each of the ribose sugar moieties is chemically modified at the 2′ position to contain either a 2′-O-methyl or a 2′-flourine (F) residue. Revusiran has twenty-two 2′-O-methyl and twenty-two 2′-F residues. These modifications confer increased resistance to endonucleases, increase the binding affinity of the duplex, and have been shown to reduce the immunostimulatory properties of siRNA molecules [7,8].

Revusiran was in development as a potential treatment for hereditary transthyretin-mediated amyloidosis (hATTR amyloidosis), a rare, life-threatening, autosomal dominant multi-systemic disease caused by mutations in the TTR gene [9]. TTR, also known as prealbumin, is a tetrameric protein produced by the liver, choroid plexus, and retina [10]. The primary role of TTR is to transport retinol (also known as vitamin A) in the circulation after binding to the retinol-binding protein (RBP): retinol complex. TTR is also a minor carrier of thyroxine [11].

Mutations in the TTR gene result in destabilization of the tetramer and dissociation into individual mutant and wild type monomers, which, subsequently, misfold. These misfolded monomers self-assemble into oligomers in the circulation and form amyloid fibrils and plaques in the extracellular space of various tissues, including the peripheral nervous system, heart, and gastrointestinal tract [12]. Individual patients with hATTR amyloidosis may manifest signs and symptoms of both polyneuropathy and cardiomyopathy [9]. Reduction of liver-derived circulating mutant and wild type amyloidgenic TTR protein using patisiran, which acts by a similar mechanism, has been shown to improve the polyneuropathy disease manifestations of hATTR amyloidosis [13].

Single and multiple doses of revusiran were generally well tolerated in healthy human subjects in phase 1 with doses of 2.5–10 mg/kg, eliciting statistically significant reductions in serum TTR protein, with mean TTR reductions of ∼90% observed with multiple dosing [14]. However, clinical development of revusiran was subsequently discontinued in the phase 3 “ENDEAVOUR” trial for patients with cardiomyopathic hATTR amyloidosis when an imbalance in mortality in the revusiran arm (500 mg [8.33 mg/kg to 60 kg patient] once daily for 5 days followed by once weekly subcutaneous [SC] administration at 500 mg) versus the placebo arm was discovered. Although no conclusive evidence was found linking the increased mortality to revusiran administration, a drug-related effect could not be excluded^*^.

An extensive nonclinical safety program (safety pharmacology, acute and repeat-dose toxicity, and genetic toxicology studies) was conducted before the initiation of phase 1. Before the start of the “ENDEAVOUR” phase 3 trial, chronic repeat-dose rat and monkey toxicity studies were completed. The chronic studies included an ultrastructural assessment of liver mitochondrial morphology to evaluate whether repeated exposure to revusiran and its metabolites resulted in mitochondrial toxicity as was reported for some nucleoside and nucleotide analog drugs, including another fluorinated compound fialuridine (1-(2-deoxy-2-fluoro-β-D-arabinofuranosyl)-5-iodouracil; FIAU) [15–19]. Revusiran had an acceptable nonclinical safety profile, and the results from these studies supported the dose and schedule that were utilized in phase 3.

Before the early termination of the “ENDEAVOUR” phase 3 trial, a 2-year rat carcinogenicity study had been initiated; this study was completed to obtain additional nonclinical safety information surrounding long-term repeated exposure to revusiran in rats with additional assessments of metabolite exposure and mitochondrial toxicity [20]. The results from the nonclinical safety and carcinogenicity assessments of revusiran are reported herein.

## Materials and Methods

### Compound

Revusiran was supplied by Althea Technologies, Inc. (San Diego, CA) or Alnylam Pharmaceuticals, Inc. (Cambridge, MA) and was characterized in compliance with good manufacturing practice or good laboratory practice as applicable. Revusiran is pharmacologically active in monkeys but not in rodents. A rodent-active surrogate (AD-59206) was supplied by Alnylam Pharmaceuticals, Inc. Stock solutions (200 mg/mL) of revusiran or AD-59206 in Sterile Water for Injection, United States Pharmacopeia (USP) were diluted with 0.9% Sodium Chloride for Injection as necessary to obtain the appropriate concentrations for dose administration.

### Animals

CByB6F1-TgRasH2 mice and wild type littermates were obtained from Taconic Farms (Germantown, NY). Sprague-Dawley rats were received from Charles River Laboratories (Kingston, NY or Raleigh, NC) (Crl:CD [SD]) or Harlan Laboratories, Inc. (Indianapolis, IN) (Hsd:Sprague-Dawley^®^ SD^®^). Cynomolgus monkeys were obtained from Covance Research Products (Alice, TX). All animals were experimentally naive. Animal studies were conducted at Alnylam Pharmaceuticals, Inc. or Covance Laboratories (Madison, WI or Greenfield, IN) according to good laboratory practice or accepted scientific and industry standards. All animal procedures were performed in full compliance with AALAC guidelines at AALAC-accredited facilities and were approved by local Institutional Animal Care and Use Committees.

### Safety pharmacology study

Revusiran was administered to 4 conscious telemeterized (Data Sciences International Dataquest^®^ OpenART^®^) male monkeys (4 years old) by SC injection at 0, 10, 30, or 100 mg/kg at a dose volume of 1.5 mL/kg on days 1, 8, 15, and 22. In this modified Latin Square design, each animal received each dose only once; all dose levels were represented on each dosing day; and every animal received a unique dosing sequence. Cardiovascular function, respiratory rate, and body temperature were recorded (PONEMAH [P3P (Ponemah Physiology Platform)] analysis system) at least 90 min before dosing and continuously for at least 19 h postdose. These data were also collected continuously at 2-h intervals from 47 to 49 and from 71 to 73 h postdose.

### Acute toxicity studies

Revusiran was administered to male and female Sprague-Dawley rats (8 weeks old) (five/sex/group) by daily SC injections at 0, 30, 100, or 300 mg/(kg·day) for 5 days at a dose volume of 1.5 mL/kg. Animals were sacrificed 1 day after the final dose. Male cynomolgus monkeys (2–3 years old) (three animals/group) received a single SC dose of revusiran at 0 or 300 mg/kg and were observed for 14 days. Acute toxicity in rats and monkeys was assessed based on clinical signs, injection site observations (monkeys), body weight, qualitative food consumption (monkeys), clinical pathology, gross examinations (rats), organ weights (rats), and microscopic pathology of the liver, spleen, kidney, lung, heart, brain, and injection sites (rats).

In monkeys, blood was collected for analysis of serum TTR concentrations, plasma complement C3a and Bb concentrations, and plasma cytokines/chemokines (CD40 ligand, G-CSF, GM-CSF, IFN-γ, IL-10, IL-12/23 (p40), IL-13, IL-15, IL-17, IL-18, IL-1RA, IL-1β, IL-2, IL-5, IL-6, IL-8, MCP-1, MIP-1α, MIP-1β, TGF-α, TNF-α, and VEGF). Serum TTR was measured by using an enzyme-linked immunosorbent assay (ELISA). Plasma complement was measured by using commercially available ELISA kits (QUIDEL^®^). Plasma cytokines were measured by using a Milliplex^®^ monkey cytokine kit and BioPlex^®^ analyzer.

### Repeat-dose toxicity studies

Repeat-dose toxicity studies were conducted in mice, rats, and monkeys. Revusiran (0, 30, 100, or 300mg/kg) or AD-59206 (30 mg/kg) was administered as weekly SC injections (10 mL/kg) to mice (9–10 weeks old) for 8 weeks; mice were sacrificed 1 day after the final dose. In rats and monkeys, revusiran was administered as a loading dose as five consecutive daily SC injections followed by weekly SC injections for 6 or 26 weeks in rats or 6 or 39 weeks in monkeys. The revusiran dose levels were 0, 30, 100 or 300mg/kg in the 6-week rat and monkey studies and were 0, 15, 30, or 100 mg/kg in the 26-week rat study and 0, 15, 75, or 200 mg/kg in the 39-week monkey study. In the 6-week rat and monkey studies, a dose volume of 1.5 mL/kg was used. In the 26- and 39-week studies, dose volumes of 2 mL/kg (rats) and 1 mL/kg (monkeys) were used.

Rats were 6–7 weeks old at the time of dosing initiation, and monkeys were 2–3 years old in the 6-week study and were sexually mature (5–6 years old) in the 39-week study. The 6- and 26-week rat and 6- and 39-week monkey repeat-dose toxicity studies each included recovery periods of 4 or 13 weeks, respectively. Assessment of toxicity was based on clinical observations, injection site observations (rats and monkeys), body weight, food consumption, ophthalmic examinations (rats and monkeys), electroretinograms (monkeys), physical examinations (monkeys), neurological examinations (monkeys), and clinical and anatomic pathology (gross examinations, organ weights, and microscopic pathology).

Electron microscopy of liver in rats and monkeys was also performed in the 26- and 39-week studies, respectively. Plasma complement (Bb and C3a) was measured by AniLytics, Inc. (Gaithersburg, MD) using a validated ELISA, and cytokines/chemokine concentrations were measured in the 6-week monkey study as described earlier with the exception that IL-4 was also measured. Male reproductive assessments were performed in the 39-week monkey study.

Blood was collected for toxicokinetics (TK) (all species), an ELISA-based anti-drug antibody assay (rats and monkeys), and determination of serum TTR concentrations (mice and monkeys), as described earlier, and vitamin A concentrations in mice and monkeys. Vitamin A (total serum retinol) was measured by AniLytics, Inc. using a high-performance liquid chromatography (HPLC)-based method. Total thyroxine was measured in mice by AniLytics, Inc. using a radioimmunoassay and reagents provided by MP Biomedicals (Solon, Ohio). In the monkey studies, thyroxine concentrations were determined by using an automated clinical chemistry analyzer. Electron microscopy was performed at Charles River Laboratories—Pathology Associates (Durham, NC) (rats) and Virginia Tech University (Blacksburg, VA) (monkeys).

### Genetic toxicology studies

Bacterial mutagenesis assays were conducted with *Salmonella* tester strains TA98, TA100, TA1535, and TA1537 and *E. coli* WP2*uvr*A with and without metabolic activation (Aroclor™1254-induced rat liver S9 homogenate; Molecular Toxicology, Inc.) at up to 5,000 μg/plate. Chromosomal aberration assays were conducted in human peripheral blood lymphocytes with and without metabolic activation at up to 500 μg/mL. A single-dose rat bone marrow micronucleus assay was conducted at revusiran doses up to 2,000 mg/kg at a 10 mL/kg dose volume; micronuclei were evaluated at 24- and 48-h postdose. All studies were conducted in compliance with good laboratory practice at Covance Laboratories (Greenfield, IN).

### Carcinogenicity study

In a 2-year carcinogenicity study in Sprague-Dawley (Crl:CD [SD]) rats, revusiran was administered as weekly SC doses of 0, 10, 30, or 100 mg/kg at a dose volume of 2 mL/kg to 60 rats/sex/group. An additional group of 60 rats/sex received weekly SC doses of 30 mg/kg AD-59206 at 2 mL/kg, a dose of the pharmacologically active rat surrogate that resulted in decreases in serum TTR that were similar to those observed in clinical studies. This surrogate arm was included to assess the possible carcinogenic effects associated with TTR lowering.

For exploratory purposes (serum chemistry, plasma lactate, electron microscopy, and bioanalysis of drug and metabolite tissue concentrations), two animals/sex/group were sacrificed at week 82. The remainder of the females and males were sacrificed at weeks 88 and 97, respectively, with three/sex/group undergoing exploratory evaluations at the terminal sacrifice.

Toxicity and carcinogenicity were evaluated based on survival, clinical observations (including assessment of palpable masses), body weight, food consumption, and clinical and anatomic pathology (gross and microscopic pathology). Blood samples were collected for TK, vitamin A, and TTR evaluation. The study was conducted in compliance with good laboratory practices at Covance Laboratories (Madison, WI). Serum TTR concentrations were measured by using ELISA at Alnylam Pharmaceuticals, Inc. using a commercially available kit for rats (ALPCO) and an internally developed assay for monkeys. Vitamin A concentrations (total serum retinol) were measured by Covance Laboratories using a HPLC-based method. Transmission electron microscopy (TEM) of selected tissues was performed by Covance Laboratories.

### Statistical analyses

Safety pharmacology data were analyzed by using repeated-measures analysis of covariance; the covariate was the mean baseline observation for each subject on each dosing day. When a significant treatment effect (*P* ≤ 0.05) was present, a Dunnett or Dunnett–Hsu *t*-test was used for group comparisons to control.

For continuous data in the repeat-dose toxicity and carcinogenicity studies, a Levene's test was applied to test for homogeneity of variance. In the case of heterogeneity of variance at *P* ≤ 0.05, rank transformation was applied. One-way analysis of variance (ANOVA) was used to analyze the data. When the result was significant at *P* ≤ 0.05, Dunnett's tests were used to compare dosed and control groups by sex at the 5.0% two-tailed probability level.

In the chromosomal aberration assay, a Cochran–Armitage test for linear trend and Fisher's exact test [21] were used to compare the percentage of treated cells with aberrations to vehicle controls. In the rat bone marrow micronucleus study, when the variances were homogeneous, ANOVA was used to evaluate untransformed proportions of cells with micronuclei per animal and polychromatic:normochromatic erythrocyte ratios. Ranked proportions were used for heterogeneous variances. If ANOVA was significant (*P* ≤ 0.05), Dunnett's *t*-test was used to compare dosed and control groups.

In the carcinogenicity study, tests to compare survival were performed with a two-sided risk for increasing and decreasing mortality with dose. Tests were performed for dose response and for each dosed group against control by using Kaplan–Meier product-limit estimates along with stratified log-rank and Wilcoxon tests using the LIFETEST procedure in SAS (Cary, NC). Tumor incidence was compared with a one-sided risk for increasing incidence with dose. Occult or non-palpable tumors were analyzed by the International Agency for Research on Cancer asymptotic fixed interval-based prevalence test [22]. Fatal and nonfatal tumors were analyzed together with a separate stratum for each. The test was implemented by using PROC MULTITEST in SAS. Unadjusted *P* values were reported for tumors.

## Results

### Safety pharmacology

Revusiran had no effects on cardiovascular or respiratory function at doses up to 100 mg/kg (highest dose tested). There were no neurological abnormalities after repeat-dose administration in monkey toxicology studies at weekly doses up to 300 mg/kg (the highest dose tested).

### Acute toxicity studies

Five consecutive daily SC doses of revusiran at 30, 100, or 300 mg/(kg·day) were well tolerated by rats. There were no toxicologically relevant changes in clinical pathology parameters, organ weights, or gross pathology findings noted on day 6. Revusiran-related microscopic findings were limited to the spleen (including minimal necrosis in the lymphoid follicles at ≥100 mg/kg and minimal vacuolation at ≥30 mg/kg) and injection sites (Table 1).

**Table 1.** Revusiran-Related Microscopic Findings Across Toxicology Species

Tissues/microscopic findings	Species							
	Rat				Monkey			Mouse
	Duration							
	Acute	6-Week	26-Week	2-Year rat carcinogenicity	Acute^a^	6-Week	39-Week	8-Week
	Doses (mg/kg)							
	30, 100, 300	30, 100, 300	15, 30, 100	10, 30, 100 or AD-59206^b^	300	30, 100, 300	15, 75, 200	30, 100, 300
	NOAEL (mg/kg)							
	300 mg/kg	30 mg/kg	30 mg/kg	N/A	300 mg/kg	300 mg/kg	200 mg/kg	300 mg/kg
Adrenal gland	No findings	No findings	Increased vacuolation, cortex ≥30 mg/kg w/full recovery	No findings	No findings	No findings	Decreased vacuolation, cortex ≥15 mg/kg w/full recovery	No findings
Injection sites	Hemorrhage, mixed cell and/or mononuclear cell infiltrates ≥30 mg/kg	Inflammatory infiltrates and vacuolation, macrophages ≥30 mg/kg w/full recovery	Vacuolation, macrophages ≥15 mg/kg w/full recovery	No findings	No findings	Vacuolation, macrophages 300 mg/kg w/full recovery	No findings	Vacuolation, macrophages ≥30 mg/kg
Kidney	No findings	Basophilic granules, tubules ≥30 mg/kg w/partial recovery	Basophilic granules, tubules ≥30 mg/kg w/partial recovery	Basophilic granules ≥10 mg/kg and tubule cell hypertrophy ≥10 mg/kg and AD-59206	No findings	No findings	No findings	No findings
Liver	No findings	Hepatocellular vacuolation ≥30 mg/kg w/partial recovery	Hepatocellular vacuolation ≥15 mg/kg and pigmented, KC at ≥30 mg/kg w/partial recovery	Hepatocellular vacuolation ≥10 mg/kg	No findings	Vacuolation, KC at ≥100 mg/kg w/partial recovery	Vacuolation, KC at ≥75 mg/kg w/full recovery	Hepatocellular vacuolation and mixed cell infiltrates ≥30 mg/kg
Lymph nodes^c^	No findings	Vacuolation, macrophages ≥30 mg/kg w/full recovery	Vacuolation, macrophages ≥15 mg/kg w/full recovery	No findings	No findings	Vacuolation, macrophages ≥30 mg/kg w/partial recovery	Vacuolation, macrophages ≥15 mg/kg w/partial recovery	No findings
Pancreas	No findings	No findings	No findings	No findings	No findings	No findings	Depletion, zymogen in males 200 mg/kg w/partial recovery	No findings
Spleen	Necrosis; follicles ≥100 mg/kg and vacuolation ≥30 mg/kg	No findings	No findings	No findings	No findings	No findings	No findings	No findings

^a^ No microscopic examination performed.

^b^ Rat-active surrogate at 30 mg/kg.

^c^ Includes axillary, mandibular, mesenteric, and/or inguinal lymph nodes.

KC, Kupffer cell(s); N/A, not applicable; NOAEL, no observed adverse effect level.

At the first (day 1) injection site, revusiran-related microscopic findings ranged from minimal to slight focal or diffuse mononuclear cell infiltrates at ≥30 mg/kg to minimal focal or diffuse mixed cell infiltrates at ≥100 mg/kg. At the final (day 5) injection site, minimal to slight focal or diffuse mixed cell infiltrates was seen in a majority of rats at ≥30 mg/kg, and occasionally accompanied by minimal to slight focal hemorrhage and/or mononuclear cell infiltrates in some rats at ≥30 mg/kg. None of the microscopic findings observed in the spleen or injections sites of rats was considered adverse.

A single SC dose of revusiran at 300 mg/kg was well tolerated in monkeys. No toxicity was observed nor were there any toxicologically relevant changes in clinical pathology parameters on day 15. There were no revusiran-related changes in complement split products C3a and Bb, or in plasma cytokines. Serum TTR protein concentrations were reduced by 80% relative to baseline on day 8 and remained reduced (90%) relative to baseline on day 15.

### Repeat-dose toxicity studies

In the 8-week dose range-finding study in mice, once weekly SC administration of revusiran (eight doses) was well tolerated at all dose levels [30, 100, or 300 mg/(kg·week)]. Microscopic findings in liver included minimally to moderately increased hepatocellular vacuolation and an increased incidence of minimal to slight mixed cell infiltrates in males at ≥30 mg/kg and females at ≥100 mg/kg (Table 1). These changes were not correlated with any changes in serum chemistry. Minimally or slightly vacuolated macrophages were noted at the final SC injection site in all of the revusiran dose groups.

Weekly SC administration of the pharmacologically active surrogate (AD-59206) at 30 mg/(kg·week) resulted in the expected reductions (80% to 85% from control) of serum TTR concentrations that were achieved by day 3 and maintained throughout the course of the study. These reductions were accompanied by reductions in circulating vitamin A (up to 80% from control) and thyroxine (up to 51% from control). There were no findings in liver at 30 mg/kg AD-59206; minimally vacuolated macrophages were present at the last SC injection site.

In the 6-week study in rats, SC administration of 30, 100, or 300 mg/kg (five daily doses followed by five weekly doses, the same schedule as that used in the initial multiple-dose clinical study [14]) revusiran was well tolerated in rats. Transient (duration typically <24 h) edema of very slight to slight severity was observed at injection sites at all doses but was most frequently observed at 300 mg/kg. At the end of the dosing phase, mean body weight, relative to controls, was significantly decreased (8%; *P* < 0.001) as was mean body weight change (19%; *P* < 0.01) in males at 300 mg/kg. The change in mean body weight did not fully recover by the end of a 4-week recovery phase.

Revusiran-related effects on clinical chemistry were mostly limited to animals dosed at ≥100 mg/kg and are summarized in Table 2. Revusiran-related changes, relative to control, included elevations in alanine aminotransferase (ALT; ≤2.3 × ), aspartate aminotransferase (AST; ≤1.4 × ), alkaline phosphatase (ALP; ≤2.7 × ), and gamma glutamyltransferase (GGT; ≤3.7 × ) and decreases in globulin (GLOB; 0.8–0.9 × ). All changes had reversed by the end of the recovery period.

**Table 2.** Mean (±Standard Deviation) Revusiran-Related Clinical Chemistry Changes in 6- and 26-Week Toxicity Studies in Rats

	6-Week toxicity study							
	End of dosing phase (day 37)^a^							
	Males				Females			
	Dose (mg/kg)							
	0	30	100	300	0	30	100	300
	No. animals/group							
	15	10	9	14	15	9	10	15
ALT (U/L)	39 ± 5.6	43 ± 7.3	65 ± 13.3^***^	89 ± 22.3^***^	32 ± 3.7	31 ± 4.5	40 ± 8.5^**^	47 ± 8.4^***^
AST (U/L)	121 ± 14.5	135 ± 19.3	144 ± 24.7	172 ± 30.1^***^	121 ± 17.7	115 ± 16.4	153 ± 65.4	149 ± 29.6^*^
ALP (U/L)	92 ± 8.4	97 ± 12.4	103 ± 9.1^*^	249 ± 34.7^***^	62 ± 8.0	56 ± 7.9	67 ± 6.4	100 ± 20.1^***^
GGT (U/L)	<3	<4	5	11	<3	<3	<3	6
Total bilirubin (mg/dL)	0.1 ± 0.00	0.1 ± 0.03	0.1 ± 0.04	0.2 ± 0.05	0.1 ± 0.04	0.1 ± 0.05	0.2 ± 0.03	0.3 ± 0.08
Cholesterol (mg/dL)	101 ± 13.5	99 ± 12.2	109 ± 12.2	128 ± 12.3^***^	88 ± 16.5	90 ± 12.2	116 ± 22.4^***^	130 ± 14.3^***^
Triglycerides (mg/dL)	22 ± 2.9	27 ± 6.4^*^	31 ± 11.3^**^	33 ± 6.3^***^	27 ± 3.3	30 ± 5.3	36 ± 5.1^***^	37 ± 4.3^***^
Albumin (g/dL)	4.3 ± 0.15	4.4 ± 0.20	4.5 ± 0.12^*^	4.5 ± 0.14^***^	4.6 ± 0.12	4.8 ± 0.26	4.8 ± 0.25	4.8 ± 0.21
GLOB (g/dL)	2.3 ± 0.19	2.1 ± 0.21^*^	2.0 ± 0.17^*^	1.9 ± 0.11^***^	2.0 ± 0.08	2.0 ± 0.05	2.0 ± 0.17	1.9 ± 0.22
Albumin:GLOB ratio	1.9 ± 0.13	2.1 ± 0.16^**^	2.2 ± 0.21^***^	2.4 ± 0.16^***^	2.3 ± 0.12	2.4 ± 0.14	2.4 ± 0.18	2.6 ± 0.28^*^
Urea nitrogen (mg/dL)	17 ± 2.0	18 ± 2.2	17 ± 1.9	16 ± 1.4	19 ± 2.5	20 ± 2.0	20 ± 2.8	16 ± 2.4^**^

	26-Week toxicity study							
	End of dosing phase (day 184)							
	Males				Females			
	Dose (mg/kg)							
	0	15	30	100	0	15	30	100
	No. animals/group							
	20	20	20	20	20	20	19	20
ALT (U/L)	44 ± 7.5	46 ± 14.6	48 ± 10.2	69 ± 27.9^***^	37 ± 8.3	34 ± 3.8	33 ± 5.4^**^	50 ± 11.5^**^
AST (U/L)	131 ± 16.5	128 ± 24.1	132 ± 21.0	161 ± 37.5^**^	134 ± 27.6	126 ± 15.1	125 ± 19.1	148 ± 24.7
ALP (U/L)	55 ± 8.3	56 ± 9.5	61 ± 10.3^*^	105 ± 16.3^***^	44 ± 12.7	46 ± 8.2	43 ± 6.2	79 ± 14.6^***^
GGT (U/L)	<3	<3	<3	<5	<3	<3	<3	<5
Total bilirubin (mg/dL)	0.1 ± 0.04	0.1 ± 0.00	0.1 ± 0.05	0.2 ± 0.05	0.2 ± 0.03	0.2 ± 0.04	0.2 ± 0.03	0.3 ± 0.05
Cholesterol (mg/dL)	147 ± 23.9	129 ± 23.0^*^	124 ± 22.1^**^	138 ± 22.9	128 ± 17.6	112 ± 20.0^*^	121 ± 18.1	148 ± 21.2^**^
Triglycerides (mg/dL)	53 ± 15.3	56 ± 16.0	59 ± 14.8	65 ± 19.9	44 ± 5.6	44 ± 5.1	43 ± 4.5	53 ± 10.9^**^
Total protein (g/dL)	7.3 ± 0.28	7.1 ± 0.22^**^	7.0 ± 0.26^***^	6.5 ± 0.27^***^	7.2 ± 0.31	7.0 ± 0.29	7.1 ± 0.24	6.7 ± 0.33^***^
Albumin (g/dL)	4.2 ± 0.16	4.2 ± 0.18	4.3 ± 0.25	4.1 ± 0.19^*^	4.7 ± 0.26	4.6 ± 0.19	4.6 ± 0.22	4.4 ± 0.22^***^
GLOB (g/dL)	3.1 ± 0.21	2.9 ± 0.23 ^**^	2.7 ± 0.14^***^	2.5 ± 0.19^***^	2.4 ± 0.17	2.4 ± 0.17	2.5 ± 0.18	2.3 ± 0.18^**^
Albumin:GLOB ratio	1.4 ± 0.10	1.4 ± 0.14	1.6 ± 0.13^***^	1.7 ± 0.15^***^	2.0 ± 0.17	1.9 ± 0.14	1.8 ± 0.18	2.0 ± 0.16
Urea nitrogen (mg/dL)	17 ± 3.6	18 ± 2.5	17 ± 1.7	19 ± 3.2^**^	19 ± 4.2	18 ± 2.9	19 ± 3.7	16 ± 2.2^*^
Creatinine (mg/dL)	0.8 ± 0.08	0.8 ± 0.08	0.8 ± 0.05	0.8 ± 0.08	0.9 ± 0.11	0.9 ± 0.06	0.8 ± 0.06	0.8 ± 0.05^**^
Phosphorus (mg/dL)	6.8 ± 0.52	6.8 ± 0.42	6.9 ± 0.47	7.3 ± 0.51^**^	6.1 ± 0.59	6.4 ± 0.69	6.4 ± 0.90	6.5 ± 0.53
Chloride (mmol/L)	102 ± 1.2	102 ± 1.3	103 ± 1.8	104 ± 1.3^***^	103 ± 1.3	104 ± 1.7	104 ± 1.5	105 ± 1.7^***^

	End of recovery period (day 276)							
	No. animals/group							
	10	10	10	10	10	9	9	10
Total protein (g/dL)	7.6 ± 0.20	7.5 ± 0.17	7.5 ± 0.15	7.2 ± 0.19^***^	7.7 ± 0.59	7.6 ± 0.33	7.4 ± 0.26	7.2 ± 0.25^*^
Albumin (g/dL)	4.1 ± 0.18	4.1 ± 0.24	4.1 ± 0.25	4.1 ± 0.22	4.9 ± 0.48	4.7 ± 0.32	4.6 ± 0.25	4.4 ± 0.23^*^
GLOB (g/dL)	3.5 ± 0.19	3.4 ± 0.23	3.3 ± 0.21	3.2 ± 0.23^**^	2.8 ± 0.15	2.9 ± 0.14	2.8 ± 0.28	2.8 ± 0.18
Urea nitrogen (mg/dL)	17 ± 2.0	18 ± 2.2	18 ± 2.6	18 ± 3.0	21 ± 3.0	17 ± 2.0^**^	19 ± 2.2	18 ± 2.1^*^
Creatinine (mg/dL)	0.7 ± 0.08	0.8 ± 0.10	0.8 ± 0.09	0.8 ± 0.07	0.9 ± 0.08	0.8 ± 0.09	0.8 ± 0.04	0.8 ± 0.09
Phosphorus (mg/dL)	6.0 ± 0.48	6.3 ± 0.41	6.5 ± 0.49	6.6 ± 0.61^*^	5.3 ± 0.93	5.5 ± 0.71	4.9 ± 0.47	6.0 ± 0.59

^a^ All findings reversed by end of 4-week recovery period.

^*^ *P* ≤ 0.05, ^**^P ≤ 0.01, ^***^P ≤ 0.001.

ALP, alkaline phosphatase; ALT, alanine aminotransferase; AST, aspartate aminotransferase; GGT, gamma glutamyltransferase; GLOB, globulin.

Revusiran-related microscopic findings were present in the kidneys (basophilic granules in proximal tubules), liver (hepatocellular vacuolation), lymph nodes (vacuolated macrophages), and injection sites (vacuolated macrophages and inflammatory infiltrates) and are summarized in Table 1. These findings had partially (kidneys and liver) or fully (lymph nodes and injection sites) reversed by the end of the recovery period. The no observed adverse effect level (NOAEL) was 30 mg/kg based on the clinical pathology changes and microscopic findings observed in liver at ≥100 mg/kg.

Findings in the 26-week study in rats (5 daily doses followed by 26 weekly doses at 15, 30, or 100 mg/kg) were similar to those observed in the 6-week repeat-dose study. Very slight to slight edema (of <24 h duration) was observed at injection sites in rats administered 100 mg/kg revusiran. At the end of the dosing phase, mean body weight, relative to controls, was significantly decreased in males at 15 mg/kg (5%, *P* < 0.05) and 100 mg/kg (10%, *P* < 0.0001). Mean body weight gain during the dosing phase was reduced in males by 8 (*P* < 0.05), 6, and 16% (*P* < 0.0001), relative to controls, at 15, 30, and 100 mg/kg, respectively. These changes in body weight were associated with sporadic occasions of reduced food consumption.

Revusiran-related changes in clinical chemistry (≤2 × elevations in ALT, AST, ALP, and GGT and 0.8–0.9 × decreases in GLOB) were similar to those observed in the 6-week study (Table 2) and reversed during the 13-week recovery period.

Microscopic findings (Table 1) included basophilic granules in the proximal tubules of the kidneys and hepatocellular vacuolation and pigmented Kupffer cells in the liver. The presence of lipid in these vacuoles was confirmed with Oil Red O staining. In addition, vacuolated macrophages were present in lymph nodes and injection sites and vacuolation of the zona fasciculata was seen in the adrenal gland. The findings in liver and kidneys were partially reversed, and the findings at the injection site and in the adrenal gland were fully reversed at the end of the recovery period.

**FIG. 1.**
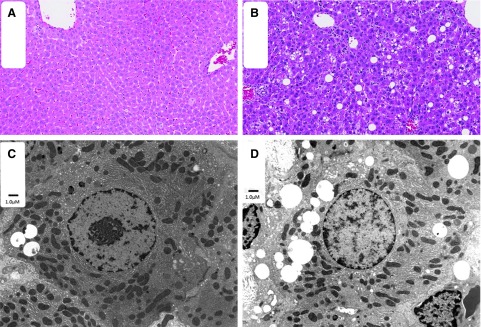
H&E staining and TEM of the liver of control and revusiran-treated rats. Minimal vacuolation is present in the liver of control **(A)** rats. Increased severity of hepatocellular vacuolation is present in the liver of high-dose **(B)** rats. Variably sized vacuoles are present in cytoplasm of hepatocytes in both control **(C)** and high-dose **(D)** male rats by TEM. There is no evidence of mitochondrial changes in hepatocytes. H&E, hematoxylin and eosin; TEM, transmission electron microscopy.

The ultrastructural examination of the liver revealed hepatocellular and sinusoidal cell (interpreted to be Kupffer cells) vacuolation at all dose levels, including controls. Hepatocellular vacuoles were noted at similar incidence but at an increasing severity (size/number) in males at ≥15 mg/kg and in females at ≥30 mg/kg when compared with controls (Fig. 1). Hepatocellular vacuoles remained in males, and Kupffer cell vacuoles remained in both sexes at the end of the 13-week recovery period. No revusiran-related ultrastructural effects were observed in mitochondria, lysosomes, or nuclei (Table 3). Given the serum chemistry changes and light microscopic correlates in liver at 100 mg/kg, the NOAEL was 30 mg/kg.

**Table 3.** Ultrastructural Findings with Revusiran and AD-59206

	Study type/duration		
	39-Week monkey toxicity	26-Week rat toxicity	2-Year rat carcinogenicity
	Dose (mg/kg)		
	15, 75, 200	15, 30, 100	10, 30, 100 mg/kg or AD-59206^a^
Specimens evaluated	Liver from main phase only (ie, no recovery evaluation)	Liver from main and recovery phases	Dorsal root ganglion (lumbar 4–5), heart, liver, skeletal muscle (soleus), and sural nerve
Ultrastructural findings	Liver:	Liver:	Liver:
	Increased KC hypertrophy and enlarged secondary lysosomes ≥75 mg/kg	Hepatocellular and KC vacuolation ≥15 mg/kg w/partial recovery	Hepatocytes: Increased incidence/severity of lipid vacuoles, lysosomes with lipofuscin, and elongated, enlarged and/or ring-shaped or cup-shaped mitochondria ≥10 mg/kg and AD-59206.
	Increased secondary lysosomes @200 mg/kg		Increased smooth endoplasmic reticulum at ≥10 mg/kg (males)
			Skeletal muscle:
			Enlarged and/or elongated mitochondria ≥10 mg/kg and increased smooth endoplasmic reticulum at 100 mg/kg (females)

^a^ Rat transthyretin surrogate at 30 mg/kg.

In the 6-week study in monkeys, revusiran was well tolerated at all dose levels (five daily doses followed by five weekly doses of 30, 100, or 300 mg/kg). Transient (<24 h) very slight to slight edema and very slight erythema were noted at the SC injection site at all dose levels. Revusiran-related clinical pathology changes were confined to reversible increases in ALP (≤3.4 × ) (Table 4). No effects on plasma complement or cytokines were noted.

**Table 4.** Mean (±Standard Deviation) Revusiran-Related Clinical Chemistry Changes in 6- and 39-Week Toxicity Studies in Monkeys

	6-Week toxicity study							
	Males				Females			
	Dose (mg/kg)							
	0	30	100	300	0	30	100	300
	No. animals/group							
	5	5	5	5	5	5	5	5
ALP (U/L)	Pre-study (week 2)							
	582 ± 182.2	517 ± 151.0	680 ± 118.8	702 ± 260.7	442 ± 60.6	428 ± 82.9	489 ± 146.5	513 ± 76.0
	Day 6							
	447 ± 122.0	582 ± 240.7	1,182 ± 343.4^*^	1,536 ± 559.6^***^	373 ± 64.6	485 ± 114.6	891 ± 302.6^***^	1,284 ± 337.3^***^
	Day 37^a^							
	551 ± 140.3	623 ± 209.6	818 ± 186.0	1,076 ± 596.2	433 ± 80.9	522 ± 104.6	819 ± 278.5^*^	852 ± 194.5^**^

	39-Week toxicity study							
	Males				Females			
	Dose (mg/kg)							
	0	15	75	200	0	15	75	200
	No. animals/group							
	6	6	6	6	6	6	6	6
ALP (U/L)	Pre-study (week 2)							
	403 ± 103.2	340 ± 46.3	309 ± 84.6	293 ± 57.6	178 ± 48.2	180 ± 39.8	166 ± 50.0	154 ± 30.3
	Pre-study (week 1)							
	421 ± 110.1	335 ± 30.7	308 ± 92.2	314 ± 50.9	176 ± 48.0	174 ± 48.1	166 ± 48.0	142 ± 29.4
	Day 275^b^							
	316 ± 92.9	284 ± 62.9	344 ± 89.3	459 ± 113.6^*^	172 ± 38.5	183 ± 30.2	251 ± 58.6	250 ± 58.0^*^

^a^ All findings reversed by end of 4-week recovery period.

^b^ All findings reversed by end of 13-week recovery period.

^*^ *P* ≤ 0.05, ^**^P ≤ 0.01, ^***^P ≤ 0.001.

Revusiran-related microscopic findings included minimal Kupffer cell vacuolation in liver and minimally to slightly vacuolated macrophages in lymph nodes (ie, axillary, inguinal, mandibular, and mesenteric) at ≥100 mg/kg (Table 1). Increased vacuolation (minimal) was also present in a few scattered macrophages at the injection site at the terminal necropsy in two females at 300 mg/kg and considered test article related. The findings in liver and lymph nodes had partially recovered at the end of a 4-week recovery period whereas the injection site findings had completely resolved.

**FIG. 2.**
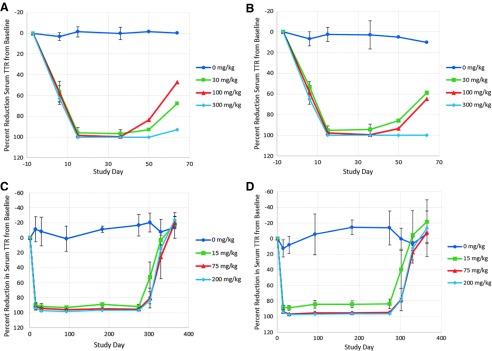
Mean (±SD) percent reduction of serum transthyretin from baseline in a 6-week or 39-week monkey study. Male **(A)** and female **(B)** cynomolgus monkeys received five daily doses of revusiran on study days 1–5 followed by once weekly injection on study days 8, 15, 22, 29, and 36. Serum TTR concentrations were determined by ELISA (LLOQ = 0.0015 μg/mL). For these analyses, values <LLOQ were assigned values of ½ LLOQ (0.00075 μg/mL). *n* = 5/sex/group (days 7, 6, 15 [predose], and 36 [predose]) and *n* = 2/sex/group (days 50 and 64). Male **(C)** and female **(D)** cynomolgus monkeys received five daily doses of revusiran followed by once weekly injection, for 39 weeks, starting on study day 8. Serum TTR concentrations were determined by ELISA (LLOQ = 1.14 ng/mL). *n* = 6/sex/group (study days 1, 15, 29, 92, 183, and 274 [all predose]) and *n* = 3/sex/group (days 302, 330, and 365). ELISA, enzyme-linked immunosorbent assay; LLOQ, lower limit of quantitation; SD, standard deviation; TTR, transthyretin.

At all dose levels, revusiran elicited expected reductions in serum TTR concentrations (≥95% from baseline) by day 15, which were maintained throughout the remainder of the study (Fig. 2). TTR concentrations partially recovered (47%–68% from baseline) at 30 and 100 mg/kg but remained reduced (≥95% from baseline) at 300 mg/kg at the end of the recovery period. Decreases in circulating vitamin A (88%–93% from baseline) and thyroxine (26%–50%) were observed at the end of the dosing phase at all dose levels, which partially or fully resolved by the end of the 4-week recovery period (Table 5). Despite these sustained decreases in vitamin A, ophthalmologic and microscopic examinations of the eyes were normal. The NOAEL was 300 mg/kg, the highest dose tested.

**Table 5.** Mean (±Standard Deviation) Revusiran-Related Vitamin A and Thyroxine Changes in 6- and 39-Week Toxicity Studies in Monkeys

	6-Week toxicity study							
	Males				Females			
	Dose (mg/kg)							
	0	30	100	300	0	30	100	300
	No. animals/group							
	5	5	5	5	5	5	5	5
Vitamin A (μg/mL)	Pre-study (week 2)							
	0.58 ± 0.090	0.60 ± 0.105	0.61 ± 0.068	0.61 ± 0.087	0.69 ± 0.172	0.58 ± 0.175	0.54 ± 0.175	0.58 ± 0.214
	Day 6							
	0.49 ± 0.085	0.20 ± 0.063^**^	0.19 ± 0.028^**^	0.18 ± 0.036^**^	0.49 ± 0.138	0.22 ± 0.066^***^	0.20 ± 0.076^***^	0.18 ± 0.044^***^
	Day 37							
	0.35 ± 0.080	0.07 ± 0.014^**^	0.05 ± 0.005^***^	0.04 ± 0.005^***^	0.38 ± 0.086	0.07 ± 0.018^**^	0.06 ± 0.012^***^	0.04 ± 0.005^***^
	Day 66^a^							
	0.60	0.24	0.32	0.14	0.67	0.31	0.29	0.16
Thyroxine (μg/dL)	Pre-study (week 2)							
	5.7 ± 1.10	5.0 ± 1.16	5.3 ± 0.66	5.4 ± 1.30	5.1 ± 0.43	6.9 ± 1.05	5.6 ± 0.98	5.3 ± 1.22
	Day 6							
	5.3 ± 0.97	3.5 ± 0.77^*^	5.0 ± 0.78	4.6 ± 1.32	5.0 ± 1.10	5.0 ± 0.70	4.5 ± 0.93	5.1 ± 1.63
	Day 37							
	5.3 ± 0.93	2.5 ± 0.78^***^	4.1 ± 0.65^*^	4.0 ± 0.50^*^	4.6 ± 1.47	4.5 ± 1.10	3.6 ± 1.13	3.9 ± 0.87
	Day 66^a^							
	5.6	4.9	5.7	4.6	4.1	5.4	5.5	5.2

	39-Week toxicity study							
	Males				Females			
	Dose (mg/kg)							
	0	15	75	200	0	15	75	200
	No. animals/group							
	6	6	6	6	6	6	6	6
Vitamin A (μg/mL)	Pre-study (week 1)							
	0.52 ± 0.288	0.49 ± 0.215	0.51 ± 0.190	0.43 ± 0.205	0.22 ± 0.096	0.26 ± 0.052	0.21 ± 0.048	0.20 ± 0.016
	Day 92							
	0.43 ± 0.127	0.09 ± 0.031^**^	0.08 ± 0.013^***^	0.07 ± 0.012^***^	0.47 ± 0.184	0.19 ± 0.115	0.09 ± 0.014^***^	0.08 ± 0.012^***^
	Day 183							
	0.35 ± 0.077	0.05 ± 0.014^***^	0.04 ± 0.012^***^	0.02 ± 0.004^***^	0.30 ± 0.067	0.05 ± 0.018^**^	0.02 ± 0.010^***^	0.02 ± 0.005^***^
	Day 275							
	0.31 ± 0.059	0.07 ± 0.016^**^	0.05 ± 0.017^***^	0.04 ± 0.009^***^	0.30 ± 0.092	0.06 ± 0.021^**^	0.03 ± 0.010^***^	0.02 ± 0.005^***^
	Day 366							
	0.42 ± 0.051	0.45 ± 0.191	0.38 ± 0.058	0.52 ± 0.049	0.49 ± 0.090	0.52 ± 0.085	0.41 ± 0.070	0.67 ± 0.135
Thyroxine (μg/dL)	Pre-study (week 2)							
	5.9 ± 1.23	4.8 ± 1.13	5.5 ± 2.06	4.6 ± 1.46	5.3 ± 0.84	6.7 ± 1.03	6.7 ± 1.43	5.8 ± 0.92
	Pre-study (week 1)							
	6.1 ± 1.10	4.9 ± 1.02	5.0 ± 1.34	4.3 ± 0.90	5.7 ± 1.52	6.3 ± 1.21	5.8 ± 0.82	6.1 ± 1.40
	Day 92							
	5.8 ± 1.40	4.2 ± 1.23	3.8 ± 1.25^*^	3.4 ± 0.74^**^	5.5 ± 1.27	4.6 ± 1.05	5.3 ± 3.19	4.4 ± 0.76
	Day 183							
	5.2 ± 0.90	3.8 ± 0.88^*^	3.2 ± 0.67^**^	3.4 ± 0.90^**^	4.8 ± 1.07	4.2 ± 0.99	3.9 ± 0.90	4.3 ± 1.16
	Day 275							
	5.9 ± 1.01	3.7 ± 1.17^**^	3.7 ± 0.62^**^	4.7 ± 1.22	6.0 ± 1.28	5.5 ± 1.88	4.5 ± 0.82	4.7 ± 0.93
	Day 366							
	5.1 ± 1.63	4.9 ± 0.83	5.0 ± 1.05	5.2 ± 1.12	4.9 ± 1.64	5.3 ± 0.34	5.1 ± 0.43	6.2 ± 0.55

^a^ Standard deviation not calculated (*n* = 2/sex/group).

^*^ *P* ≤ 0.05, ^**^P ≤ 0.01, ^***^P ≤ 0.001.

Similar findings were noted in the 39-week study in monkeys that employed slightly lower doses (5 daily doses followed by 39 weekly doses at 15, 75, or 200 mg/kg) than those tested in the 6-week study. Slight edema at injection sites was noted 2 and 4 h postdose at 200 mg/kg that had resolved by 24 h. Mean body weight, relative to controls, was reduced by 27% (*P* < 0.01) in the high dose males; mean body weight gain at 200 mg/kg in these males was 10% compared with 34.5% in the control males (*P* < 0.01). There were no revusiran-related effects on male reproductive assessments (sperm motility, sperm density, sample weights, or total sperm counts). Reversible revusiran-related elevations in ALP of ≤1.5 × were observed (Table 4).

At the end of the dosing phase, vacuolated Kupffer cells were noted at 200 mg/kg as were vacuolated macrophages in lymph nodes (ie, axillary, inguinal, mandibular, and mesenteric) at ≥15 mg/kg (Table 1). In addition, decreased vacuolation of the adrenal gland was observed at ≥15 mg/kg as was zymogen depletion in the exocrine pancreas at 200 mg/kg. At the end of the 13-week recovery period, the findings in liver and the adrenal gland had completely resolved; findings in pancreas (200 mg/kg) and lymph nodes (≥75 mg/kg) had partially resolved.

Ultrastructural examination of the liver from the main study animals revealed Kupffer cell hypertrophy with increased numbers and enlarged secondary lysosomes in both sexes administered ≥75 mg/kg of revusiran (Table 3). In both sexes at 200 mg/kg, there was an increase in secondary lysosomes within hepatocytes. No mitochondrial changes were noted. None of the microscopic or ultrastructural findings was considered adverse.

Pharmacological reductions in serum TTR (≤98% from baseline) (Fig. 2), vitamin A (≤95% from baseline) (Table 5), and thyroxine (≤36% from baseline) (Table 5) were observed at all dose levels and had reversed by the end of the recovery period. There were no effects of these vitamin A reductions on ophthalmic examinations, electroretinograms, or histopathology of the eye. The NOAEL was 200 mg/kg, the highest dose tested.

### Genetic toxicology studies

The bacterial mutagenesis assay was negative in all 5 tester strains (in presence and absence of S9 metabolic activation) at up to 5,000 μg/plate. In human peripheral blood lymphocytes, revusiran was not clastogenic at up to 500 μg/mL with or without S9 metabolic activation. Single SC doses of revusiran at 500, 1,000, or 2,000 mg/kg did not induce micronucleus formation in the bone marrow of Sprague-Dawley rats.

### Carcinogenicity

In a 2-year rat study, once weekly SC administration of revusiran at 10, 30, 100 mg/kg or AD-59206 (a rat surrogate) at 30 mg/kg to 60 rats/sex/group had no effect on survival. Females were terminated during week 88 and males were terminated during week 97 based on the number of surviving control animals (*N* = 20). At the time of necropsy, the number of surviving females was 20, 16, 21, 17, or 30 and the number of surviving males was 20, 23, 23, 27, or 21 for controls and those administered 10, 30, or 100 mg/kg revusiran or 30 mg/kg AD-59206, respectively.

Males administered ≥10 mg/kg revusiran or 30 mg/kg AD-59206 gained less weight than controls (5%, not significant (NS); 13%, *P* < 0.05; or 21%, *P* < 0.0001) for 10, 30, or 100 mg/kg revusiran and 15% (*P* < 0.01) for 30 mg/kg AD-59206) from weeks 1 to 94. As a result, mean body weight at week 94 was significantly less than control at 30 (11%, *P* < 0.05) and 100 mg/kg (18%, *P* < 0.0001) revusiran and 30 mg/kg AD-59206 (13%, *P* < 0.01). Females at 100 mg/kg revusiran also gained less weight (23%, *P* < 0.05) than controls from weeks 1 to 86; mean body weight at week 86 in these females was significantly lower (16%, *P* < 0.05) than controls. In males at 100 mg/kg revusiran, the effects on body weight were generally correlated with reduced food consumption.

Week 52 hematology results for revusiran and AD-59206 revealed no evidence of leukemogenic potential. In tissues, including liver, the primary organ of uptake, kidney, the primary organ of excretion, and the SC injection sites, no revusiran- or AD-59206-related neoplastic or hyperplastic findings occurred. There were a few occasions of statistically significant trends and/or group-wise differences in revusiran-treated animals for some common tumor types (Table 6). However, none of these observations was considered related to revusiran in either sex because they lacked a dose response, are common neoplasms in Sprague-Dawley rats [23,24], or because their incidence remained within historical control data range for the laboratory.

**Table 6.** Summary of Statistically Significant Tumor Results

Males			
Tissue and lesion	Rare or common	Test	Unadjusted *P* value
Skin/Subcutis	Common	Trend	0.0418 (LR)
B-Fibroma			0.0493 (W)
Skin/Subcutis	Common	Trend	0.0079 (LR)
M-Fibrosarcoma			0.0085 (W)
Skin/Subcutis	Common	Trend	0.0058 (LR)
B-Fibroma/M-Fibrosarcoma			0.0065 (W)
		High vs. control	0.0214 (LR)
			0.0348 (W)

Females			
Tissue and lesion	Rare or common	Test	Unadjusted *P* value
Mammary gland	Common	Low vs. control	0.0468 (LR)
M-Carcinoma		Mid vs. control	0.0026 (LR)
Pituitary	Common	Trend	0.00295 (P)
B-Adenoma, pars distalis		High vs. control	0.0484 (P)
Pituitary	Common	Trend	0.0465 (P)
B-Adenoma, pars distalis/M-Carcinoma, pars distalis			
Skin/subcutis	Common	Trend	0.0103 (LR)
B-Papilloma, squamous cell			0.0125 (W)

LR, log-rank test; P, Peto test; W, Wilcoxon test.

Non-neoplastic microscopic findings that were related to revusiran (all dose levels) or AD-59206 at 30 mg/kg included an increased incidence and severity of slight to marked hepatocellular vacuolation. In kidney, an increased incidence of minimal to moderate basophilic granules and tubule cell hypertrophy was noted at all dose levels of revusiran and at 30 mg/kg AD-59206.

Exploratory TEM did not reveal any revusiran- or AD-59206-related ultrastructural changes in heart, dorsal root ganglion, or sural nerve (Table 3). At termination, revusiran- and AD-59206-related ultrastructural changes in liver at all dose levels included an increased incidence/severity of lipid vacuoles, lysosomes with lipofuscin, and elongated and/or ring-shaped or cup-shaped mitochondria. An increase in smooth endoplasmic reticulum was also observed in males only at ≥10 mg/kg revusiran. In skeletal muscle, revusiran-related changes at all dose levels included enlarged and/or elongated mitochondria and an increase in smooth endoplasmic reticulum in females at 100 mg/kg revusiran. In mitochondria, there were no ultrastructural changes in the cristae in either skeletal muscle or liver [20].

AD-59206, the rat active surrogate molecule, elicited the expected pharmacological reductions in serum TTR concentrations (>99%), relative to control, whereas there were no differences in the revusiran-treated groups. Likewise, reductions in thyroxine (62%–84%) and vitamin A (86%–96%), relative to control, were observed in males and females treated with AD-59206 but not in those treated with revusiran. There were no neoplastic or hyperplastic findings that were correlated with these reductions in TTR, thyroxine, and vitamin A.

### Animal to human exposure multiples

Plasma pharmacokinetics were estimated in patients after 10 doses of revusiran at 7.5 mg/kg in the phase 2 trial. At 100 mg/kg in the 8-week dose range-finding mouse toxicology study (the only dose in which plasma TK were assessed), and at the NOAEL in rats (30 mg/kg) and monkeys (200 mg/kg) in the chronic toxicology studies, respective C_max_ values were 23×, 1× and 26× the human C_max_ value and respective AUC_last_ values were 15×, 1.6× and 106× the human AUC_last_ value as shown in Table 7.

**Table 7.** Plasma Exposure to Revusiran at No Observed Adverse Effect Level and Animal to Human Exposure Multiples in Mice, Rats, and Monkeys

Study	NOAEL (mg/kg)	C_max_*^a^* [μg/mL (multiple*^b^*)]	AUC_last_*^a^* [μg × h/mL (multiple*^b^*)]
8-Week toxicity in mice	100^c^	29.6 (23)	88.9 (15)
26-Week toxicity in rats	30	1.21 (1)	9.70 (1.6)
39-Week toxicity in monkeys	200	33.2 (26)	645 (106)

^a^ Mean of C_max_ and AUC_last_ for males and females (combined) after final dose administration.

^b^ Human C_max_ (1.263 μg/mL) and AUC_last_ (6.103 μg × h/mL) from patients (*n* = 3) after final (10th) dose administration at 7.5 mg/kg.

^c^ Mouse C_max_ and AUC_last_ values were available at mid-dose (100 mg/kg) only.

AUC_last_, area under the concentration-time curve from dosing to the last measurable concentration; C_max_, maximum observed concentration.

## Discussion

There were no cardiovascular or respiratory safety pharmacology concerns for revusiran in monkeys. An *in vitro* human ether-à-go-go-related gene assay with revusiran was not conducted because revusiran, similar to a therapeutic protein, is a relatively large molecule (∼16 kDa). The molecular size of revusiran would preclude interaction with ion channels. Further, the triantennary GalNAc ligand of revusiran binds the hepatocyte ASGPR to facilitate cellular uptake in hepatocytes. In a quantitative whole-body autoradiography (QWBA) study in rats, minimal radioactivity was recovered in the heart, suggesting minimal uptake into cardiac tissue. Moreover, there were no revusiran-related neurological examination results in repeat-dose studies in monkeys. Little to no uptake of radioactivity was noted in brain in the rat QWBA study, which suggests that revusiran does not cross the blood**–**brain barrier.

In the acute toxicity study in rats, revusiran-related findings were noted in spleen and at the SC injection sites. The minimal necrosis and vacuolation in spleen observed acutely were not observed in subsequent repeat-dose toxicity studies in rats. The spleen is not typically a target organ of toxicity for GalNAc-siRNA conjugates [25]. The injection site findings in rats were recapitulated in subsequent repeat-dose toxicity studies in rats. Although no histopathology was performed in the single-dose acute toxicity study in monkeys, there were no clinical pathology changes indicative of toxicity nor was there any evidence of immune stimulation; no complement activation (as gauged by plasma split products Bb and C3a) or elevations in plasma cytokines were observed.

In repeat-dose studies in rats and monkeys, revusiran-related deficits in body weight and/or body weight gain, relative to control, were observed at 300 mg/kg in male rats in the 6-week study and at all dose levels (15, 30, or 100 mg/kg) in male rats in the 26-week study. In the 39-week study in monkeys, reduced body weight and body weight gain was observed in males at 200 mg/kg. In both species, these changes were sporadically associated with reduced food consumption. Although the cause of these body weight deficits in revusiran-treated males has not been elucidated, there were no apparent effects on the health and well-being of the animals and these changes, therefore, were not considered adverse.

Revusiran-related findings were present in liver, lymph nodes, kidney (rats only), and injection sites. Most of these findings (eg, lipid-filled hepatocellular vacuoles [rats only] and vacuolated and/or pigmented Kupffer cells in liver, vacuolated macrophages in lymph nodes, basophilic granules in renal proximal tubules, and vacuolated macrophages and inflammatory infiltrates at SC injection sites) are considered to be class effects associated with SC administration of siRNA-GalNAc conjugates to nonclinical species [25].

In rats, the changes in liver were accompanied by modest (≤4 × control) elevations in several liver enzymes. Transient elevations in GGT in rats and ALP in rats and monkeys were observed at ≥75 mg/kg but were not associated with any histopathological evidence of cholestatic hepatobiliary injury or toxicologically relevant increases in total bilirubin. The Kupffer cell and macrophage vacuolation had no apparent functional effects on the immune system.

The microscopic findings at the SC injection sites were not associated with any adverse injection site reactions; transient very slight to slight edema, sometimes accompanied by very slight erythema, were the only injection site changes observed in rats and monkeys.

The increased incidence and severity of vacuoles in the zona fasciculata of the adrenal gland in male rats likely represented a nonadverse exacerbation of a normal background finding; it was also observed in controls and had no correlative clinical pathology changes. In contrast, decreased vacuolation in the zona fasciculata of the adrenal gland was observed in both sexes of monkeys at the end of the chronic study. The minimal to slight zymogen depletion in the pancreas of high-dose male monkeys was not considered adverse and was not accompanied by any other pancreatic changes. A clear relationship of the adrenal and pancreatic findings to revusiran is difficult to establish; ASGPR expression is highly specific to hepatocytes [26] and, as such, there was little to no radiolabeled revusiran detected in the adrenal gland or pancreas in the QWBA study in male rats.

All of these findings were partially or fully reversible in rats and monkeys and with exception of the hepatocellular vacuolation in rats, which were not considered adverse. In rats, the NOAEL of 30 mg/kg in both the 6- and 26-week studies was based on liver findings that were correlated with the serum chemistry changes at higher doses.

FIAU is a fluorinated antiviral nucleoside analogue developed for the treatment of hepatitis B that caused multisystem toxicity, including liver failure, after repeat dosing [27]. Ultrastructural examination of the liver revealed swollen, misshapen mitochondria with decreased numbers of cristae.

Revusiran contains twenty-two 2′F modified nucleotides. An ultrastructural examination of liver was conducted as part of the pathology evaluation in the 6-month rat and 9-month monkey studies to evaluate whether revusiran had any potential ultrastructural effects on mitochondria. No mitochondrial changes were noted in either study after chronic administration of revusiran. Two important chemical differences between FIAU and the 2′F-modified nucleotides in revusiran is that FIAU comprises 2′F arabinose sugar instead of the 2′deoxy-2′F-ribose contained in revusiran and a uracil base with iodine at the 5 position that is not present in the 2′F uridines in revusiran.

The siRNA in revusiran is completely homologous with the TTR mRNA target sequence in humans and cynomolgus monkeys and, thus, is pharmacologically active in monkeys. It has no activity in mice or rats. In addition to revusiran, a rodent-active surrogate, AD-59206, was included in the 8-week repeat dose range-finding study in CByB6F1-TgRasH2 mice and their wild type littermates. This surrogate was also included in the 2-year rat carcinogenicity study. As discussed by Kornbrust *et al.* [28], surrogate (analog) molecules and their human drug counterparts have different nucleotide sequences and, therefore, can potentially have different toxicity profiles. This was not the case for AD-59206, at doses that resulted in reductions in serum TTR in rodents that were similar to those observed in clinical studies.

In the 8-week repeat dose range-finding study in mice, AD-59206 elicited the expected reductions in circulating TTR, vitamin A, and thyroxine without any apparent effects on tissues (eg, eye and thyroid) due to secondary pharmacology. In a similar manner, the revusiran-mediated reductions in TTR, vitamin A, and thyroxine in monkeys had no secondary effects on the histopathology of the thyroid, eye, ophthalmic examination results, or electroretinograms. These results are consistent with reports of humans with RBP/TTR mutations who have life-long low circulating vitamin A concentrations and remain in good health [29] and TTR knockout mice [30] that do not manifest signs of vitamin A deficiency, suggesting that there are compensatory transport mechanisms for vitamin A.

Revusiran was nongenotoxic in a standard battery of *in vitro* and *in vivo* genetic toxicity assays conducted according to the International Council for Harmonisation guidelines. Neither revusiran at ≤100 mg/kg nor AD-59206 at 30 mg/kg was carcinogenic in a 2-year rat bioassay. There were no revusiran or AD-59206-related effects on survival. The reductions in body weight gain, relative to controls, observed at all revusiran doses and at 30 mg/kg AD-59206 in males and at a high dose in females were consistent with those observed in previous toxicity studies in rats. As in those studies, the reduced body weight gain did not impact survival or have any apparent overall health effects.

The non-neoplastic findings of hepatocellular vacuolation in liver and basophilic granules in kidney tubules were similar to those observed in repeat-dose toxicity studies in rats and did not significantly progress in severity over the course of the long-term carcinogenicity study. An additional renal finding (tubular hypertrophy) had not been observed in the shorter toxicity studies in rats.

The carcinogenicity study was ongoing when the phase 3 “ENDEAVOUR” clinical trial was halted. Several exploratory endpoints were added to the study, including assessment of interim and terminal clinical chemistry, plasma lactate, and TEM evaluation of mitochondria in liver, nerve, dorsal root ganglion, heart, and skeletal muscle. Consistent with the results from earlier rat toxicity studies, there were no adverse revusiran or AD-59206 changes in clinical chemistry, including plasma lactate concentrations; minimally higher ALP activity (2.5–4.9 × control) was present at 100 mg/kg.

Although enlarged and ring-shaped mitochondria were observed in liver and skeletal muscle, they did not structurally resemble the swollen hepatic mitochondria with disorganized cristae observed in patients treated with FIAU and there were no apparent toxic effects of revusiran or AD-59206 on the mitochondria [20]. Despite the sustained AD-59206-mediated reductions in circulating TTR, vitamin A, and thyroxine over the entire course of the study, there was no evidence of increased tumorigenesis in these rats. A 26-week carcinogenicity assay in TgRasH2 mice was planned but was canceled after the clinical development of revusiran was halted.

As the intended patient population for revusiran was primarily elderly males with hATTR amyloidosis with cardiomyopathy, a rat embryo-fetal development study was the only reproductive toxicity study included in the nonclinical development plan. Clinical development of revusiran was halted before this study was conducted.

In conclusion, comprehensive toxicology studies were conducted that provided adequate exposure multiples, relative to humans. Rats were the most sensitive nonclinical species and exhibited reduced body weight gain along with modest serum chemistry changes that correlated with microscopic liver findings. In addition to liver, revusiran-related findings were consistently observed in kidney, lymph nodes, and SC injection sites and were partially or fully reversible, not considered to be adverse, and consistent with findings frequently observed with other siRNA-GalNAc conjugates.

Revusiran was not genotoxic or carcinogenic in a 2-year bioassay in rats. In monkeys, the pharmacologically relevant species, revusiran-mediated reductions in TTR, vitamin A, and thyroxine did not result in any adverse effects. A rodent-active surrogate was included in the 2-year rat carcinogenicity study and it was demonstrated that long-term sustained reductions in circulating TTR, vitamin A, and thyroxine did not affect tumorigenesis.

Taken together, these results demonstrated a favorable nonclinical safety profile for revusiran that did not predict the mortality imbalance subsequently observed in the clinic. Potential reasons for the lack of nonclinical predictivity regarding the outcome of the “ENDEAVOUR” phase 3 trial may include species differences, age, and/or overall health (ie, normal healthy animals in nonclinical studies vs. elderly amyloidosis patients with cardiomyopathy and advanced heart failure in the “ENDEAVOUR” phase 3 trial).

## Footnotes

^*^ Judge DP, AV Kristen, M Grogan, MS Maurer, RH Falk, M Hanna, J Gillmore, P Garg, AK Vaishnaw, *et al*. (2019). Phase 3 multicenter study of revusiran in patients with hereditary transthyretin-mediated (hATTR) amyloidosis with cardiomyopathy (ENDEAVOUR). Cardiovasc Drug Ther.
